# Revisiting Concurrent Radiation Therapy, Temozolomide, and the Histone Deacetylase Inhibitor Valproic Acid for Patients with Glioblastoma—Proteomic Alteration and Comparison Analysis with the Standard-of-Care Chemoirradiation

**DOI:** 10.3390/biom13101499

**Published:** 2023-10-10

**Authors:** Andra V. Krauze, Yingdong Zhao, Ming-Chung Li, Joanna Shih, Will Jiang, Erdal Tasci, Theresa Cooley Zgela, Mary Sproull, Megan Mackey, Uma Shankavaram, Philip Tofilon, Kevin Camphausen

**Affiliations:** 1 Radiation Oncology Branch, Center for Cancer Research, National Cancer Institute, National Institutes of Health (NIH), 9000 Rockville Pike, Building 10, CRC, Bethesda, MD 20892, USAtheresa.cooleyzgela@nih.gov (T.C.Z.); uma@mail.nih.gov (U.S.); philip.tofilon@nih.gov (P.T.);; 2 Computational and Systems Biology Branch, Biometric Research Program, Division of Cancer Treatment and Diagnosis, National Cancer Institute, National Institutes of Health, Rockville, Maryland 20850, USA; zhaoy@mail.nih.gov (Y.Z.); limingc@mail.nih.gov (M.-C.L.); jshih@mail.nih.gov (J.S.)

**Keywords:** glioma, radiation, proteomic, valproic acid, HDAC inhibitor

## Abstract

Background: Glioblastoma (GBM) is the most common brain tumor with an overall survival (OS) of less than 30% at two years. Valproic acid (VPA) demonstrated survival benefits documented in retrospective and prospective trials, when used in combination with chemo-radiotherapy (CRT). Purpose: The primary goal of this study was to examine if the differential alteration in proteomic expression pre vs. post-completion of concurrent chemoirradiation (CRT) is present with the addition of VPA as compared to standard-of-care CRT. The second goal was to explore the associations between the proteomic alterations in response to VPA/RT/TMZ correlated to patient outcomes. The third goal was to use the proteomic profile to determine the mechanism of action of VPA in this setting. Materials and Methods: Serum obtained pre- and post-CRT was analyzed using an aptamer-based SOMAScan^®^ proteomic assay. Twenty-nine patients received CRT plus VPA, and 53 patients received CRT alone. Clinical data were obtained via a database and chart review. Tests for differences in protein expression changes between radiation therapy (RT) with or without VPA were conducted for individual proteins using two-sided *t*-tests, considering *p*-values of <0.05 as significant. Adjustment for age, sex, and other clinical covariates and hierarchical clustering of significant differentially expressed proteins was carried out, and Gene Set Enrichment analyses were performed using the Hallmark gene sets. Univariate Cox proportional hazards models were used to test the individual protein expression changes for an association with survival. The lasso Cox regression method and 10-fold cross-validation were employed to test the combinations of expression changes of proteins that could predict survival. Predictiveness curves were plotted for significant proteins for VPA response (*p*-value < 0.005) to show the survival probability vs. the protein expression percentiles. Results: A total of 124 proteins were identified pre- vs. post-CRT that were differentially expressed between the cohorts who received CRT plus VPA and those who received CRT alone. Clinical factors did not confound the results, and distinct proteomic clustering in the VPA-treated population was identified. Time-dependent ROC curves for OS and PFS for landmark times of 20 months and 6 months, respectively, revealed AUC of 0.531, 0.756, 0.774 for OS and 0.535, 0.723, 0.806 for PFS for protein expression, clinical factors, and the combination of protein expression and clinical factors, respectively, indicating that the proteome can provide additional survival risk discrimination to that already provided by the standard clinical factors with a greater impact on PFS. Several proteins of interest were identified. Alterations in GALNT14 (increased) and CCL17 (decreased) (*p* = 0.003 and 0.003, respectively, FDR 0.198 for both) were associated with an improvement in both OS and PFS. The pre-CRT protein expression revealed 480 proteins predictive for OS and 212 for PFS (*p* < 0.05), of which 112 overlapped between OS and PFS. However, FDR-adjusted *p* values were high, with OS (the smallest p value of 0.586) and PFS (the smallest *p* value of 0.998). The protein PLCD3 had the lowest *p*-value (*p* = 0.002 and 0.0004 for OS and PFS, respectively), and its elevation prior to CRT predicted superior OS and PFS with VPA administration. Cancer hallmark genesets associated with proteomic alteration observed with the administration of VPA aligned with known signal transduction pathways of this agent in malignancy and non-malignancy settings, and GBM signaling, and included epithelial–mesenchymal transition, hedgehog signaling, Il6/JAK/STAT3, coagulation, NOTCH, apical junction, xenobiotic metabolism, and complement signaling. Conclusions: Differential alteration in proteomic expression pre- vs. post-completion of concurrent chemoirradiation (CRT) is present with the addition of VPA. Using pre- vs. post-data, prognostic proteins emerged in the analysis. Using pre-CRT data, potentially predictive proteins were identified. The protein signals and hallmark gene sets associated with the alteration in the proteome identified between patients who received VPA and those who did not, align with known biological mechanisms of action of VPA and may allow for the identification of novel biomarkers associated with outcomes that can help advance the study of VPA in future prospective trials.

## 1. Introduction

Glioblastoma (GBM) is the most common and the most aggressive brain tumor [1]. The current standard of care involves maximal surgical resection followed by concurrent radiation therapy (RT) and temozolomide (TMZ), followed by adjuvant TMZ [2]. The prognosis in GBM remains poor, with an overall survival (OS) of less than 30% at two years. Several therapies [3] have been studied to improve outcomes beyond the chemoirradiation (CRT) Stupp regimen, which was the first to result in improvement in OS by adding TMZ to RT [2]; however, while some benefits were described in some, none of these attempts have made an appreciable impact on OS. Valproic acid (VPA) has been one of the agents studied in this context, given its use as an antiepileptic agent in glioma patients who often present with seizures. Its activity as an HDAC inhibitor [4,5,6], its use as an antiepileptic agent [7,8,9], its association with improvement in survival [7,8,10,11,12,13], as well as its value given the cost of care [14], have made VPA an attractive agent of study [15,16,17,18] and the subject of several reviews [19,20,21]. Several studies have revealed potential anti-tumor effects via several cancer hallmark pathways, including angiogenesis, DNA repair, stemness, cellular reprogramming, apoptosis, and the epithelial-to-mesenchymal transition [22,23,24,25], including synergism in conjunction with TMZ [26,27]. The precise mechanisms of action that may underlie possible improvements in outcomes have, however, remained ill-defined even as a relationship between VPA dose [9,22,28,29] and duration [30] emerged that may well explain discordant outcome results in meta-analyses [11,31] given the use of seizure dose VPA as compared to high-dose VPA [17] and treatment duration as well as evolving seizure management over time transitioning from VPA to increase use of Levetiracetam [9,31,32,33]. The addition of VPA to concurrent RT/TMZ in patients with newly diagnosed GBM in our previous phase II trial was well tolerated, resulted in a favorable toxicity profile, had no late effects (neurological, pain, and blood/ bone marrow toxicity and mostly grade 1/2 and only two grade 3/4 toxicities), and improved outcomes (median OS 29.6 months (range: 21–63.8 months) [15,16,17]. The analysis of the proteomic alteration signatures post chemoirradiation in conjunction with OS was previously described [34]. In this study, we aimed to determine whether differential alteration in proteomic expression pre- vs. post-completion of concurrent chemoirradiation (CRT) is present with the addition of VPA to CRT, and if present, link proteomic alteration to both OS and PFS, and the biological mechanisms of action of VPA via prognostic and predictive protein signals.

## 2. Materials and Methods

### 2.1. Patients

Twenty-nine patients who received concurrent valproic acid (VPA) were compared to 53 patients who received CRT alone. All patients had pathology-proven GBM (diagnosed 2005–2013) and were enrolled on NCI NIH IRB-approved protocols. The patients who received concurrent high-dose VPA were treated on the open-label, NCI NIH phase 2 study (NCT00302159). To be included in this analysis in the VPA class, the patients needed to have received ≥1 week of VPA as per the original analysis [17] and have biospecimen obtained amenable to proteomic analysis. Patients who received CRT alone needed to have documented tissue diagnosis of glioblastoma and have received standard of care concurrent chemoirradiation defined as 59.4–60 Gy in 30–33 fractions with concurrent TMZ on natural history protocols (NCT00027326, NCT00083512). Blood biospecimens obtained before and after CRT completion were included in the study. In the patients who received VPA, this was initiated one week before the first day of RT at 10 to 15 mg/kg/day and subsequently increased up to 25 mg/kg/day over the week before RT. Analysis of their initial outcomes and late toxicity was previously published [15,16,17]. Serum samples were screened using the multiplexed, aptamer-based approach (SOMAScan^®^ assay) to measure the relative concentrations of 7596 protein targets (7289 human) for changes in expression using approximately 150 ul of serum [35,36]. Clinical data (age, gender), tumor characteristics (location, MGMT methylation status), management-related factors (extent of resection), radiation therapy volumes (GTV T1, GTV T2), recursive partitioning analysis score (RPA) [37], and outcomes (PFS, OS) were obtained or derived (RPA) from the protocol database and electronic health record with GTV T1, GTV T2, generated per ICRU report 83 [38] obtained from the radiation therapy treatment planning (contoured on the T1 gadolinium sequence of the MRI scan employed for RT planning per standard guidelines).

### 2.2. SomaLogic SOMAScan^®^ Assays

Serum samples were obtained before initiation of CRT (average seven days, range (0 to 23)) and following completion of CRT (average seven days, range (−1 to 30)) with the time between pre- and post-sample acquisition averaging 49 days (range 27–83 days). Following the acquisition, samples were frozen at −80 for an average of 3442 days (range 800–5788 days) and then defrosted and screened using the aptamer-based SOMAScan^®^ proteomic assay technology for changes in the expression of 7000+ protein analytes [35,36]. SOMAScan^®^ data were filtered to remove non-human and non-protein targets, resulting in 7289 aptamers targeting 6386 unique gene symbols. RFU values reported by SOMAScan^®^ were log2-transformed.

### 2.3. Data Process

There were 7596 proteins on the chip. We selected 7289 human proteins for the subsequent analyses. There were 82 patients with data before CRT (PRE) and post-completion of CRT (COT (Completion of Treatment)). Log base 2 (COT/PRE) represented the protein expression change between after-treatment and pre-treatment conditions.

### 2.4. Class Comparisons

Tests for differences in clinical characteristics and protein expression changes between CRT with or without VPA were conducted using two-sided *t*-tests, considering *p*-values of <0.05 as significant. Adjustment for age, sex, and other clinical covariates was performed separately when appropriate. The Benjamini and Hochberg method was used to estimate the false discovery rate [39]. Hierarchical clustering of significant differentially expressed proteins was carried out using BRB-ArrayTools Dynamic Heatmap [40].

### 2.5. Gene Set Enrichment Analysis

Fifty Cancer Hallmark gene sets were downloaded from MSigDB. Gene Set Enrichment analyses were performed in BRB-ArrayTools [40]. The Kolmogorov–Smirnov (KS) tests are applied separately to each of the 50 gene sets. A gene set is considered significant if its corresponding KS re-sampling *p*-value is below the specified threshold (*p*-values < 0.05). GSEA with adjustments for age and sex were performed.

### 2.6. Survival Analysis

In the previous iteration of proteomic sample analysis in this cohort, univariate and multivariate Cox analysis was carried out for OS [34]. This iteration aimed to examine potential differential protein alteration pre- vs. post-CRT, seeking to define protein expression between patients who received VPA and those who did not. Univariate Cox proportional hazards models were fit to test individual protein expression change levels for association with both OS and PFS. Regression coefficients from these models were tested using a two-sided Wald test, considering *p*-values < 0.05 as significant. First, we examined the association between the expression of individual proteins and survival in unadjusted and adjusted analyses. Using protein expression changes above and below 0 Kaplan–Meier survival curves were plotted using the R statistical package [41].

Two models were employed to examine whether alteration in the protein expression with the administration of VPA can provide additional survival risk discrimination to that already provided by the standard covariates and, if so, to evaluate how much risk discrimination power the protein expression can add to the clinical covariates. To test the combinations of expression changes of proteins that could predict survival, we used the Lasso Cox regression method [42,43] implemented in BRB-ArrayTools [40]. To avoid overfitting due to the initial supervised selection of proteins to define the prognostic index, we used 10-fold cross-validation. A log-rank statistic is computed to assess whether the association of expression data to survival data is statistically significant. The significance of the log-rank statistics is determined by the permutation test. In the combined model, permutation test is performed by shuffling expression profiles while preserving survival data and covariates. Cross-validated Kaplan-Meier curves and log-rank statistics are generated, yielding a *p*-value that assesses whether expression data significantly enhances risk prediction compared to covariates. Cross-validated time-dependent ROC curves were employed for the model containing standard covariates and including both standard covariates and protein expression to evaluate whether the expression data provide more accurate predictions than those provided by standard clinical covariates in the case without separate test data [44].

Based on the pre-CRT protein measurement, predictiveness curves were plotted for each significant protein that is predictive for VPA response (*p*-value < 0.005) to show the survival probability vs. the protein expression percentiles [45]. Based on the cross point of the predictiveness curves, any new samples/patients would thus be evaluated for the superior response given the addition of VPA treatment. Furthermore, Kaplan–Meier curves were generated for both VPA groups using the stratified data in the low-/high-score cohorts in OS and PFS, respectively.

**Figure 1:**
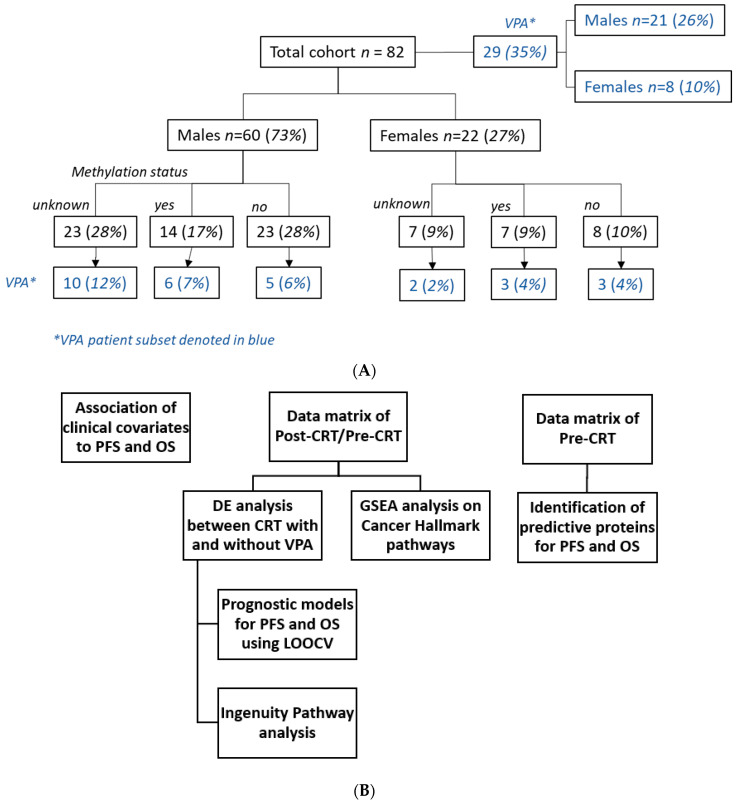
(**A**) Patient cohort breakdown by gender, methylation status and valproic acid (VPA) administration. (**B**) Data analysis workflow.

In summary, this study primarily focused on analyzing protein expression changes induced by treatment, with the exception of predictive protein identification using the pre-CRT measurement. The data analysis workflow is visually represented in Figure 1B and the models with mathematical formulas are available as supplementary materials in Supplementary File S1.

## 3. Results

### 3.1. Clinical Features Comparison between VPA and Non-VPA Cohort

Twenty-nine patients received VPA, and 53 received CRT alone (Figure 1A). MGMT methylation status was 9/29 (31%) vs. 12/53 (23%) methylated, 8/29(28%) vs. 23/53(43%) unmethylated, and 12/29(41%) vs. 18/53(34%) unknown, for VPA vs. non-VPA cohorts, respectively (Figure 1A) and MGMT methylation status was not statistically significant between the two cohorts (Table 1). VPA patients were statistically different from non-VPA patients with respect to age, extent of resection KPS, RPA, and RT technique (Table 1), with VPA patients being younger (mean age 52 vs. 58, but similar range 31–71), enriched in GTR resection status and fewer biopsies and superior KPS and RPA.

### 3.2. Clinical Factors Survival and Progression-Free Survival Analysis

In the VPA cohort, MGMT methylation status and RPA were statistically significant for OS. On univariate analysis, MGMT unmethylated status and RPA class 4 were associated with adverse outcomes (HR 3.2 and 2.86, respectively) (Supplementary Table S1). RPA was the only clinical feature statistically significant for PFS.

When combining VPA and non-VPA cohorts on Cox regression analysis (Supplementary Table S2, Figure S1) age, RPA, MGMT status, GTV T1, cortical vs. periventricular location, either hemisphere vs. bilateral disease, tumor location, and the administration of VPA were statistically significant for OS. The administration of VPA resulted in superior OS (*p* = 0.011) in Kaplan–Meier analysis (Supplementary Figure S2A). Age, BMI, RPA, MGMT status, cortical vs. periventricular location, tumor location, and the administration of VPA were statistically significant for PFS. The administration of VPA resulted in superior PFS (*p* = 0.015) in Kaplan–Meier analysis (Supplementary Figure S2B).

### 3.3. Differentially Expressed Proteins VPA vs. No VPA

**Figure 2:**
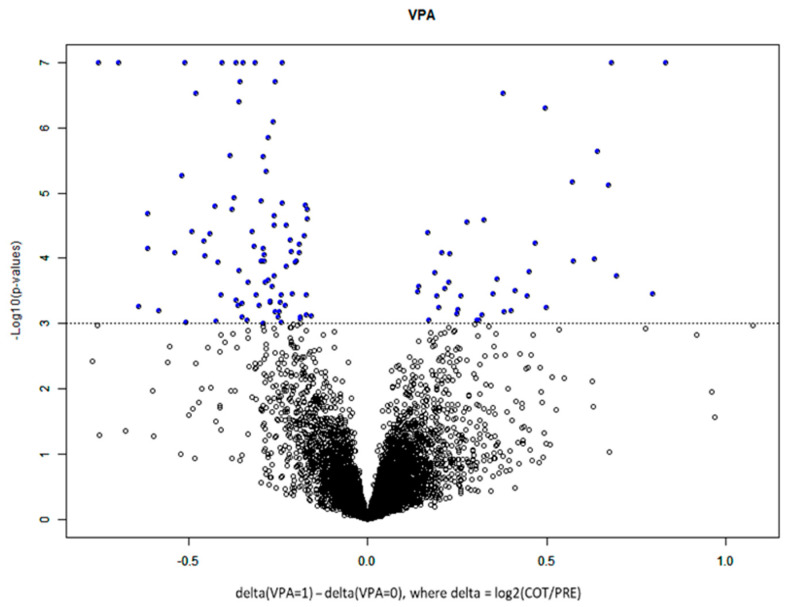
Volcano plot of class comparison of patients who received chemoirradiation (53 samples, class 0) and patients who received chemoirradiation plus VPA (29 sample, class 1) for the 7289 measured proteins using log2(cot-pre) data. Blue dots indicate the 124 differentially expressed proteins in the VPA-treated population with significance level unadjusted *p* < 0.001 and FDR-adjusted *p*-value below 0.058.

**Figure 3:**
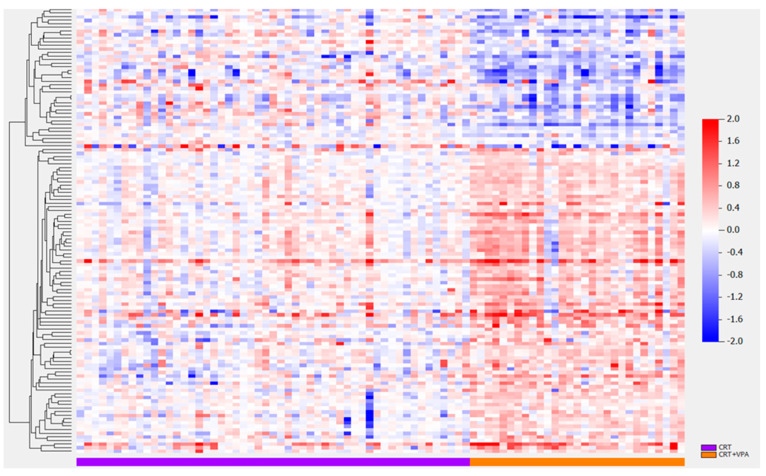
Hierarchical clustering of 124 significantly differentially expressed proteins between patients treated with CRT and concurrent VPA vs. CRT. Supplementary Figure S4A,B display the protein names for top (Figure S4A) and bottom cluster (Figure S4B), respectively.

The protein signal was not affected by the time in the freezer from collection to analysis (Supplementary Figure S3 showing a signal for proteins GALNT14 and SKP1). Differentially expressed proteins were examined between two classes: patients who received CRT alone (class 0) and patients who received CRT plus VPA (class 1) (Figure 2, Table 2). One hundred twenty-three proteins were identified that were altered pre- vs. post-CRT between the two classes. T-test results for the number of significant proteins between classes were adjusted for the impact of clinical covariates (Table 2). Similar numbers of significantly differentially expressed proteins were identified when adjusting for clinical variables compared to the unadjusted model (row #1) except for tumor location, wherein multiple categories were present with few samples in each category. Overall, this indicates that the results are globally not confounded by clinical covariates, including MGMT status (the last two rows), which are very similar (57 vs. 59) (Table 2). Hierarchical clustering of the 124 differentially expressed proteins revealed distinct proteomic clustering in the VPA-treated population (Figure 3, Supplementary Figure S4A,B display the gene names for the top (Figure S4A) and bottom cluster (Figure S4B), respectively) with significance level unadjusted *p* < 0.001 and FDR-adjusted *p*-value below 0.058 for all identified proteins (Supplementary File S2). Of the proteins statiscally significant for OS (GALNT14, CCL17, CTSV, ACP6, BMP6, SLITRK6, MSTN, NPTX1, ICAM4, SLITRK5), the top 10 displayed in Table 3 sorted in ascending order by OS FDR (all proteins displayed in Supplementary File S2), GALNT14 and CCL17 were the only two proteins with an FDR of 0.198 (Table 3). For PFS, GALNT14, ACP6, FBLN7, ALB, SLITRK5, TLNRD1, GNS, FCGRT, STAP1 and PRTG were stastically significant; the FDR, however, was 0.232 or higher for all proteins (Table 3, Supplementary File S2).

### 3.4. Survival Models for OS and PFS

**Figure 4:**
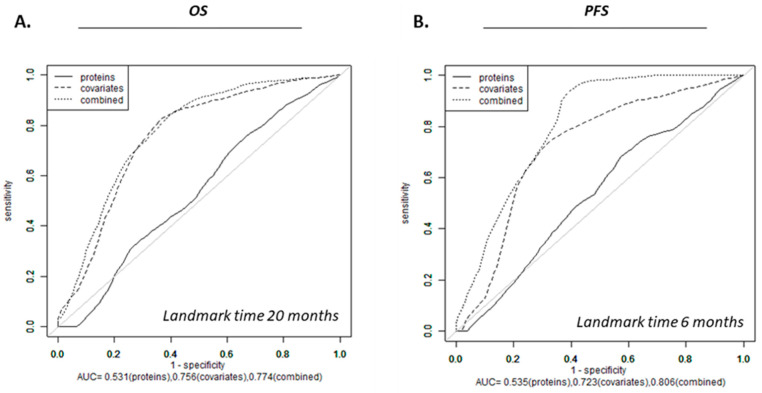
Time-dependent ROC curve for overall survival (OS) (**A**) and progression-free survival (PFS) (**B**) prediction model. (**A**) OS by proteins, covariates and combination of proteins and covariates. (**B**) PFS by proteins, covariates and combination of proteins and covariates.

**Figure 5:**
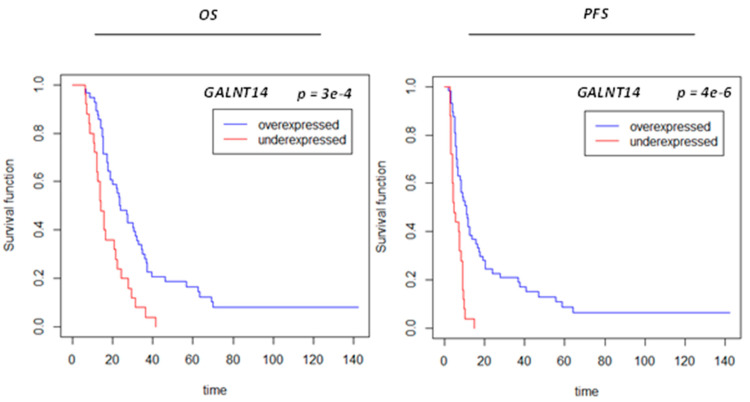
Kaplan–Meier curves for overall survival (OS) (**A**) and progression-free survival (PFS) (**B**) by difference in GALNT14 expression pre- vs. post-completion of chemoirradiation.

To evaluate whether clinical covariates can predict survival, three statistically significant clinical variables identified in the univariate analysis, i.e., age, GTV-T1, and VPA, were included in a multivariate Cox model for overall OS and PFS, respectively (Supplementary Table S2 and Figure S1). The other covariates not retained were RPA (“unknown” status was significant), MGMT status (unknown in 34% and 41% of the non-VPA and VPA cohorts, respectively), hemisphere vs. bilateral disease, and location (too few samples in each category). To examine whether protein expression provides additional survival risk discrimination to that already provided by the three clinical covariates, protein signals identified as significantly differentially expressed between VPA and non-VPA (Supplementary File S2) were included in survival models for OS and PFS and Lasso regression was used to build the survival risk prediction models. Landmark times were selected based on the clinically observed and published literature median survival and progression times for GBM [46,47,48]. Cross-validated time-dependent ROC curves for OS and PFS for landmark times of 20 months and 6 months, respectively, were generated revealing AUC of 0.0.531, 0.756, 0.774 for OS and 0.535, 0.723, 0.806 for PFS for protein expression, clinical covariates and the combination thereof, respectively, indicating improved risk discrimination power with the addition of protein expression to the clinical covariates. A more significant impact on PFS as compared to OS was observed (Figure 4). As shown in Supplementary Figure S5, the cross-validated Kaplan–Meier curves and log-rank tests indicated that protein-only models were not statistically significant for OS or PFS, clinical covariates were significant for both OS and PFS, and combined models versus covariates only model were not significant for PFS or OS (Supplemental Figure S5). GALNT14, ACP, SLITRK5 and CCL17 were associated with both OS and PFS (Table 3). Among the above four proteins, GALNT14 was retained in univariate Cox proportional hazard models with an increased pre- vs. post-CRT value associated with improved OS and PFS (Table 3, Figure 5). We experimented with a survival model employing GALNT14 without other protein signals; however, this did not improve AUC (Supplementary Figure S6).

### 3.5. Protein Signals Predictive of VPA Response

**Figure 6:**
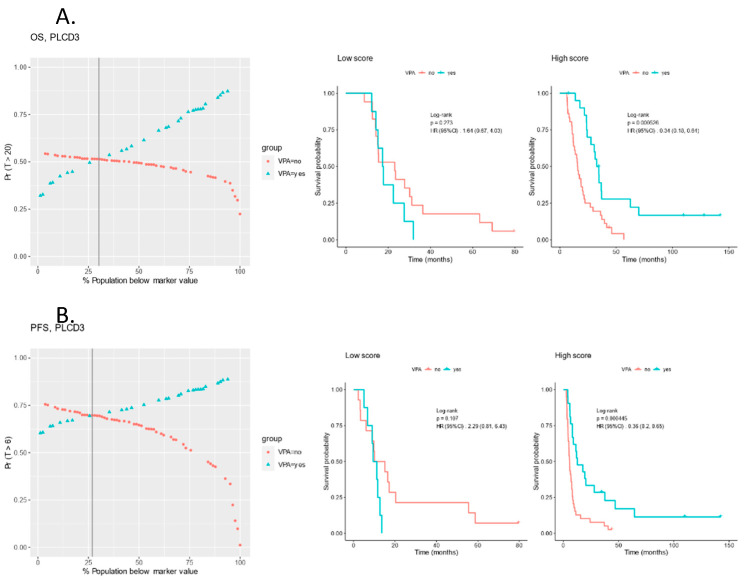
Predictiveness curves for the PLCD3 protein for (**A**) OS and (**B**) PFS and KM survival curves for high- and low-score groups. Vertical grey line indicates crossing point/threshold percentile of 30% and 27% for OS and PFS respectively.

The pre-CRT protein expression was analyzed for predictive proteins and, with *p* < 0.05, revealed 480 proteins associated with OS and 212 associated with PFS, of which 112 overlapped between OS and PFS (Supplementary File S3). PLCD3, VARS1, CYREN and KIR2DL4 emerged as predictive protein signals with *p* < 0.005 for both OS and PFS. For PLCD3, a higher score predicting superior OS and PFS with VPA administration (*p* < 0.0005) and a robust differential survival probability vs. the protein expression percentiles (Figure 6).

### 3.6. Pathway Analysis

Cancer hallmark genesets associated with VPA administration were analyzed. They identified several pathways related to the signal transduction pathways of VPA in malignancy and non-malignancy settings as well as tumor proliferation and migration, including the epithelial–mesenchymal transition (EMT), hedgehog signaling, Il6/JAK/STAT3, coagulation, NOTCH, apical junction, xenobiotic metabolism, and complement signaling (Supplementary Table S3). EMT had the most significant number of matched proteins (150).

## 4. Discussion

The ability to evaluate proteomic changes in a cohort of patients, such as the one in this study, represents a unique opportunity to analyze the impact of an additive component to the standard-of-care CRT by, in this case, examining the effect of VPA administered as part of a prospective protocol. We have previously shown that the patient population treated with concurrent VPA in this trial [17] had superior outcomes as compared to those described in the literature [15] and in modern-day trials [46,47,48], with an acceptable toxicity profile [16]. We have previously reported on the global proteomic alteration identified pre- vs. post-CRT in GBM [34]. The current analysis aims to define the effect of VPA administration on the proteome pre- vs. post-CRT in GBM compared to patients who received standard-of-care CRT alone.

In this study, we identified differential proteomic expression between patients who received CRT and those who received CRT plus VPA with 124 proteins differentially expressed, indicating that the effects of VPA provide a sufficiently enriched proteomic signal. We note that the sample size employed in this study [49,50] compares favorably with previous studies of similar scope [51], aligning with the design of similar studies that employ large-scale data [52] (Supplementary Table S4). We also note that serum sample acquisition before and after CRT is feasible despite the time range from collection to analysis (2003–2015), with time spent in storage ranging from 2 to 15 years. The proteomic signal remains robust, unaffected by storage, and significantly altered to capture intervention with VPA and CRT. The analysis of the proteomic signal revealed that protein expression was distinctly clustered in the patients who received VPA. This is interesting given that although this is a large-scale proteomic data set, it still represents only a tiny subset of the proteome overall, and it is presumably impacted by innumerable competing factors: clinical factors, medications, and other factors that have yet to be proteomically defined.

We found that in the OS and PFS models, the proteomic signal conferred additive risk discrimination power as compared to the proteome or the clinical data in isolation, and this was more pronounced for PFS in the context of VPA treatment. This is logical given that the signal alteration resulted from pre- vs. post-CRT proteome, which would more likely impact PFS since it represents a “closer” outcome endpoint to sample acquisition compared to OS, which occurs later. OS is also subject to multiple other factors, including further resection, additional systemic management, patient performance status, and comorbidities. It should also be noted that the ability to capture and interpret progression as an endpoint is limited and, thus, PFS remains flawed with clinical information limited by inconsistencies (clinical, radiographic, or both); hence, presumably, PFS remains relatively insensitive. Based on this analysis, employing proteomic alteration with validation in larger cohorts may be possible to identify more robust signals and better predict progression.

The GALNT14 protein was identified in both protein and protein-plus-covariate models, and was significant for both OS and PFS, with an increase following CRT and VPA treatment, measured as an improvement in OS and PFS and thus prognostic for both. GALNT14 is a member of the polypeptide N-acetylgalactosaminyltransferase (GALNT) family comprised of enzymes that catalyze mucin-type O-glycosylation of proteins. Alterations in components of this family have been associated with several hallmarks of cancer, including migration, proliferation, and treatment resistance [53,54]. GALNT14 has been expressed in multiple cancers altering several biological functions, and was recently described as an emerging marker capable of predicting outcomes [53]. Previous data have shown that GALNT14 correlates with Apo2L/TRAIL sensitivity in pancreas cancer, non-small-cell lung cancer, and melanoma cell lines, with overexpression increasing response to treatment by leveraging O-glycosylation to mediate apoptosis-initiating protease caspase-8 [55], decreasing resistance to apoptosis. The impact of altered O-glycosylation by members of the GALNT family is wide-ranging, as recently described in genome-wide analyses, and under- and over-expressed genes likely exhibit different effects on different cancers [54]. The role of GALNT14 in glioma has not been described, nor has its role as part of the human proteome and, thus, requires more research and validation. However, alternation in this protein in our study appears to correlate to the administration of VPA and survival. VPA, as noted, impacts multiple signaling pathways [21], and its mechanism as a radiation modifier has yet to be fully understood. Our study is the first to connect GALNT14, an emerging oncologic marker, to VPA administration, GBM biology, and the human proteome. Additional markers were identified (Supplemental material File 4). CTSV (Cathepsin V), ACP6 (Acid phosphatase 6, lysophosphatidic), BMP6 (Bone morphogenetic protein 6), MSTN (Myostatin), SLITRK6 (SLIT and NTRK like family member 6), ICAM4 (Intercellular adhesion molecule 4 (Landsteiner-Wiener blood group), NPTX1 (Neuronal pentraxin 1) and SLITRK5 (SLIT and NTRK like family member 5). All these proteins have relevance to cancer (Supplemental material File 4), while BMP6, MSTN, SLITRK5 and 6 and ICAM4 and NPTX1 have specific relevance to GBM with, in the case of SLTGK5 and 6 and NPTX1 having been linked to neural tissue and neurodegeneration respectively.

PLCD3 emerged as one of the most statistically significant predictive proteins by *p*-value for both OS and PFS, in addition to VARS1, CYREN, KIR2DL4 all with *p* < 0.005 for both OS and PFS. PLCD3 had a higher value before CRT associated with the improved outcomes with the administration of VPA, and this bears further investigation, as do the additional predictive proteins identified in this study. Several predictive proteins identified have connections to known pathways in cancer, including PLCD3 (phospholipase C delta3), VARS1 (Valyl-tRNA synthetase 1), CYREN (Cell cycle regulator of NHEJ), KIR2DL4 (Killer cell immunoglobulin-like receptor 2DL4) and SKP1(S-phase kinase-associated protein 1), and have a direct link to GBM [56,57] (Supplemental File 4). PLCD3 connects to PI3K (Phosphatidylinositol-4,5-bisphosphate 3-kinase) and EGFR, which are both critical in GBM, with PI3K signaling exhibiting heterogeneous signaling that is the subject of ongoing investigation [58,59]. Notably VARS1 may relate to seizure presentation and management in the context of GBM, while CYREN likely relates to radiation and chemotherapy managements given its association with non homologous end joing DNA repair pathways. KIR2DL4 has been implicated in cancer in relationship to the immune microenviroment and is subject to evolving mangement avenues for targeted cancer immunotherapy (Supplemental File 4). SKP1 is the most significant predictive protein for OS and a transcription regulator with significant connections to molecular pathways in cancer and GBM specifically [60,61]. In its interaction with β-transducin repeat-containing E3 ubiquitin-protein ligase (β-TrCP), itself a substrate recognition subunit for the Skp1-Cullin1-F-box protein E3 ubiquitin ligase, it has been implicated in tumorigenesis and regulation of pathways with β-TrCP suppressing progression and cell migration in glioma. It has also been reported to induce chromosome instability via cyclin E1 in other cancers [62]. Additional proteins, including AGR3, APPL1, CCL17, CLU, and FBLN7, are all supported by data in GBM, with their role an ongoing research interest [63,64,65,66,67].

**Figure 7:**
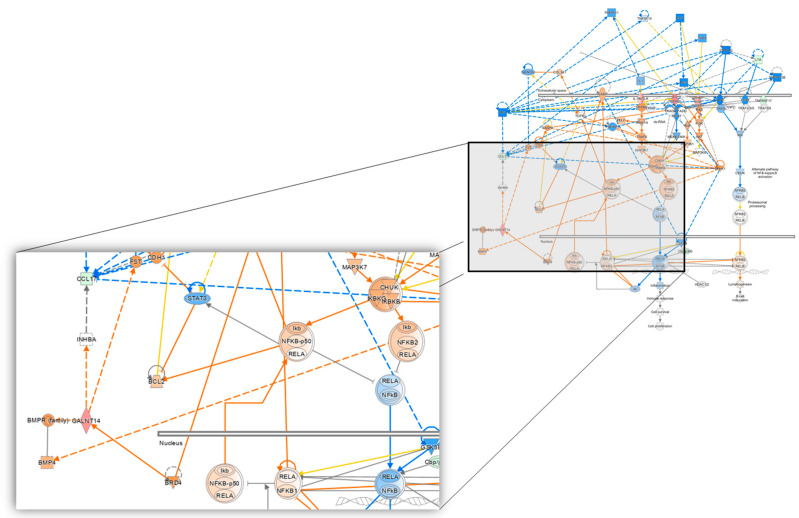
Ingenuity Pathway Analysis (IPA) overlaying the alteration of 124 differentially expressed proteins onto the NF-κB and STAT3 pathways showcasing the effect of GALNT14 and CCL17. (QIAGEN Inc., https://www.qiagenbioinformatics.com/products/ingenuitypathway-analysis, accessed on 24 July 2023 [71]).

The statistically significant proteins identified in this study and hallmark gene sets associated with the alteration in the proteome observed between patients who received CRT plus VPA and those who received CRT alone align with known biological mechanisms of action of VPA as an HDACi [21], including effects on apoptosis [28,68], DNA repair and damage response [23], signaling via Notch and TGF [30], BMP [69,70], NF-κB and STAT3 [6] (Figure 7, Supplementary Figure S7) [71]. Understanding the effect of VPA by exploiting observed proteomic alteration may allow for understanding the benefit observed with its administration in glioma patients and identifying novel biomarkers that can help advance the study of VPA in future prospective trials to improve outcomes.

This study’s limitations include the small cohort and retrospective nature of the study, given the comparison with patients who did not receive VPA and were not treated on trial. The outcomes were superior to real-world data and thus may not be representative. The data set was missing MGMT in 37% of patients, and IDH status was unavailable. The data can not be validated with additional cohorts since no such cohorts are treated with high-dose VPA and available proteome, and large-scale proteomic data are lacking. Moreover, while adjusting for clinical covariates in the prognostic models, the potential interactions among these covariates were not incorporated. This omission could introduce bias if significant interactions exist and are associated with survival outcomes. Future directions include building prediction models wherein we will explore several techniques to mitigate class imbalance [72]. There are also additional impacts of other factors on the proteome, including steroids, thrombosis, and medications that are not captured in the clinical classification, and these can impact the analysis and interpretation. We also do not know if the changes in the proteome stem from the tumor or from normal tissues, or if the changes observed are representative of cause or effect.

## 5. Conclusions

Differentially expressed proteins were identified in a large-scale proteomic panel carried out before and post-completion of CRT with the addition of high-dose VPA. GALNT14 and CCL17 were identified as potential prognostic markers based on their expression changes between pre- and post-treatment proteomic profiles. Additionally, PLCD3, SKP1, VARS1, CYREN, and KIR2DL4 were found to be potentially predictive biomarkers solely from the expression levels in the pre-treatment proteomic profile. Those findings were supported by existing data in cancer with several proteins specifically reported on in GBM with growing associations to treatment resistance or response. The predictive and prognostic ability following measurement in serum in GBM patients with the addition of VPA to the standard-of-care CRT is novel, as is the identification of several proteins that, collectively or individually, may be explored as biomarkers. Predictive markers may point to biological risk groups that may benefit from administering VPA to result in superior outcomes. Given these findings, VPA benefits from additional studies in prospective trials.

## Figures and Tables

**Table 1:** Clinical covariate comparison of patients who received concurrent CRT vs. patients who received CRT plus VPA.

VPA Administration	No	Yes	*p*-Value
**Total cohort n = 82 (*%*)**	53 (*64.6*)	29 (*35.4*)	
**Age (mean (SD))**	58.06 (10.54)	52.34 (9.60)	*0.018*
*Age Range*	*31–70*	*31–71*	
**Gender = Male (%)**	39 (*73.6*)	21 (*72.4*)	*1*
**Location**			
**Periventricular or Cortical = Periventricular (%)**	22 (*41.5*)	6 (*20.7*)	*0.097*
**Hemisphere**			*0.614*
**Left**	23 (*43.4*)	15 (*51.7*)	
**Right**	29 (*54.7*)	14 (*48.3*)	
**Both**	1 (*1.9*)	0 (*0.0*)	
**Extent of Resection**			*0.04*
**GTR**	15 (*28.3*)	16 (*55.2*)	
**STR**	31 (*58.5*)	12 (*41.4*)	
**Biopsy**	7 (*13.2*)	1 (*3.4*)	
**MGMT status**			*0.361*
**methylated**	12 (*22.6*)	9 (*31.0*)	
**unmethylated**	23 (*43.4*)	8 (*27.6*)	
**unknown**	18 (*34.0*)	12 (*41.4*)	
**KPS**			*0.014*
**60–80**	14 (*26.4*)	3 (*10.3*)	
**90**	24 (*45.3*)	12 (*41.4*)	
**100**	10 (*18.9*)	14 (*48.3*)	
**Unknown**	5 (*9.4*)	0 (*0.0*)	
**RPA**			*0.019*
**3**	5 (*9.4*)	9 (*31.0*)	
**4**	29 (*54.7*)	17 (*58.6*)	
**5**	16 (*30.2*)	3 (*10.3*)	
**Unknown**	3 (*5.7*)	0 (*0.0*)	
**RT volumes**			
**GTV T1 (cc)**			*0.437*
**<20 cc**	13 (*24.5*)	11 (*37.9*)	
**20–40 cc**	19 (*35.8*)	9 (*31.0*)	
**>40 cc**	21 (*39.6*)	9 (*31.0*)	
**GTV T2 (cc)**			*0.152*
**<10 cc**	8 (*15.1*)	0 (*0.0*)	
**10–50 cc**	13 (*24.5*)	8 (*27.6*)	
**50–100 cc**	15 (*28.3*)	8 (*27.6*)	
**>100 cc**	17 (*32.1*)	13 (*44.8*)	
**RT Technique**			*<0.001*
**3D**	15 (*28.3*)	9 (*31.0*)	
**IMRT**	18 (*34.0*)	20 (*69.0*)	
**Arc**	20 (*37.7*)	0 (*0.0*)	

**Table 2:** The number of significant proteins in different class comparisons adjusted by clinical covariates.

Class Comparison	Class 0	Class 1	Adjusted by	# of Significant Proteins (*p* < 0.001)
VPA	VPA = 0 (53)	VPA = 1 (29)		124
VPA	VPA = 0 (53)	VPA = 1 (29)	age (>65)	109
VPA	VPA = 0 (53)	VPA = 1 (29)	sex	128
VPA	VPA = 0 (53)	VPA = 1 (29)	GTV-T1 (median)	127
VPA	VPA = 0 (53)	VPA = 1 (29)	Resection type	134
VPA	VPA = 0 (53)	VPA = 1 (29)	KPS	110
VPA	VPA = 0 (53)	VPA = 1 (29)	RPA	108
VPA	VPA = 0 (53)	VPA = 1 (29)	Radiation Technique	89
VPA	VPA = 0 (53)	VPA = 1 (29)	GTV-V2 (median)	125
VPA	VPA = 0 (53)	VPA = 1 (29)	Infiltration	104
VPA	VPA = 0 (52)	VPA = 1 (29)	Hemisphere	120
VPA	VPA = 0 (51)	VPA = 1 (28)	BMI (median)	114
VPA	VPA = 0 (53)	VPA = 1 (29)	Location	83
VPA with known MGMT	VPA = 0 (35)	VPA = 1 (17)		57
VPA with known MGMT	VPA = 0 (35)	VPA = 1 (17)	MGMT	59

**Table 3:** Top 10 protein signals significant for overall survival (OS) and progression-free survival (PFS) by *p*-value based on the results from 124 significantly differentially expressed proteins from the comparison of patients treated with CRT plus VPA vs. CRT alone.

Symbol	Name	EntrezID	Fold-Change	Mean log2(COT/PRE)	OS *p*-Value	OS FDR	OS HR	PFS *p*-Value	PFS FDR	PFS HR
**CCL17**	*C-C motif chemokine ligand 17*	6361	1.620	−0.004	0.003	0.198	1.513	0.024	0.232	1.363
**GALNT14**	*polypeptide N-acetylgalactosaminyltransferase 14*	79623	0.780	0.186	0.003	0.198	0.407	0.004	0.232	0.388
**CTSV**	*ch* *a* *thepsin V*	1515	1.160	−0.088	0.009	0.285	3.647	0.024	0.232	3.031
**ACP6**	*acid phosphatase 6, lysophosphatidic*	51205	0.810	0.184	0.011	0.285	0.408	0.007	0.232	0.378
**BMP6**	*bone morphogenetic protein 6*	654	0.850	0.134	0.012	0.285	0.344	0.027	0.240	0.369
**MSTN**	*myostatin*	2660	0.850	0.089	0.019	0.304	0.277	0.093	0.339	0.424
**SLITRK6**	*SLIT and NTRK like family protein 6*	84189	0.770	0.116	0.021	0.304	0.494	0.194	0.496	0.729
**ICAM4**	*intercellular adhesion molecule 4 (Landsteiner-Wiener blood group)*	3386	1.240	−0.267	0.027	0.304	2.002	0.039	0.246	1.806
**NPTX1**	*neuronal pentraxin 1*	4884	0.870	0.050	0.029	0.304	0.286	0.280	0.504	0.615
**SLITRK5**	*SLIT and NTRK like family protein 5*	26050	0.720	0.229	0.031	0.304	0.542	0.013	0.232	0.475

## Data Availability

The data pertaining to this study has been made available as supplementary material to this manuscript.

## Supplementary Materials

The following supporting information can be downloaded at https://www.mdpi.com/article/10.3390/biom13101499/s1. File S1: Survival models with mathematical formulas; File S2: The 124 differentially expressed proteins in the VPA-treated population; File S3: Pre-CRT protein expression analyzed for predictive proteins showing the 112 proteins overlapped between OS and PFS; File S4: Additional identified proteins in the reanalysis post data correction; Figure S1: Forest plot for covariates for overall survival (OS) and progression free survival (PFS); Figure S2: KM curves for OS (A) and PFS (B) for the administration of VPA; Figure S3: Protein signal vs. time in freezer from collection to analysis in days for. (A) GALNT14, statistically significant for OS and PFS in Cox analysis and survival. (B) SKP1, predictive for response to VPA in prediction model; Figure S4: Figure 3 displayed as the two separate zoom-in figures with gene names to represent the top (A) and bottom (B) clusters of proteins in the original heatmap; Figure S5: Kaplan Meier curves for survival (OS) (A-C) and progression free survival (PFS) (D–F) with patient population risk stratified (high(red), low (blue)) by protein expression (A,D), covariates (B,E) and combination of protein expression and covariates (C,F) Protein data is based on the results from 124 significantly differentially expressed proteins from class comparison between patients treated with CRT and concurrent VPA vs CRT. Figure S6: Time-dependent ROC curve for overall survival (OS) (A) and progression free survival (PFS) (B) prediction model based on GALNT14; Figure S7: GALNT14 and CCL17, the two proteins associated with OS with FDR 0.104 with potential function between two major signaling pathways driven by TGF and NF-κB. (QIAGEN Inc., https://www.qiagenbioinformatics.com/products/ingenuitypathway-analysis) accessed on 24 July 2023 [71]; Table S1: Patient characteristics table and cox regression analysis of clinical covariates in the VPA cohort; Table S2: Cox analysis for overall survival (OS) and progression free survival (PFS) with hazard ratio and p-value for VPA and no VPA patients.; Table S3: Cancer Hallmark GeneSets associated with Valproic acid (VPA) administration; Table S4: Results of online sample size calculation tool to calculate the minimum sample size for PFS and OS. References [73,74,75,76,77,78,79,80,81,82,83,84,85,86,87,88,89,90,91,92] were cited in Supplementary Materials.

## Abbreviations

CRT	Concurrent Chemoirradiation
EMT	Epithelial–Mesenchymal Transition
GBM	Glioblastoma
GTV T1	Gross Tumor Volume on T1 Gadolinium-Enhanced MRI Sequence
GTV T2	Gross Tumor Volume on T2 FLAIR Signal Sequence
MGMT	O6-Methylguanine-DNA Methyltransferase
OS	Overall survival
PFS	Progression-Free Survival
RPA	Recursive Partitioning Analysis
RT	Radiation Therapy
TMZ	Temozolomide
VPA	Valproic Acid
WHO	World Health Organisation
