# Supplementary material for: Revisiting Concurrent Radiation Therapy, Temozolomide, and the Histone Deacetylase Inhibitor Valproic Acid for Patients with Glioblastoma—Proteomic Alteration and Comparison Analysis with the Standard-of-Care Chemoirradiation
Source: Biomolecules. 2023 Oct 10;13(10):1499. doi: 10.3390/biom13101499 (PMC10604983; doi:10.3390/biom13101499)
Supplement: Supplementary file 1 [file biomolecules-13-01499-s001.zip › Supplementary figures and tables.pdf]

Supplemental

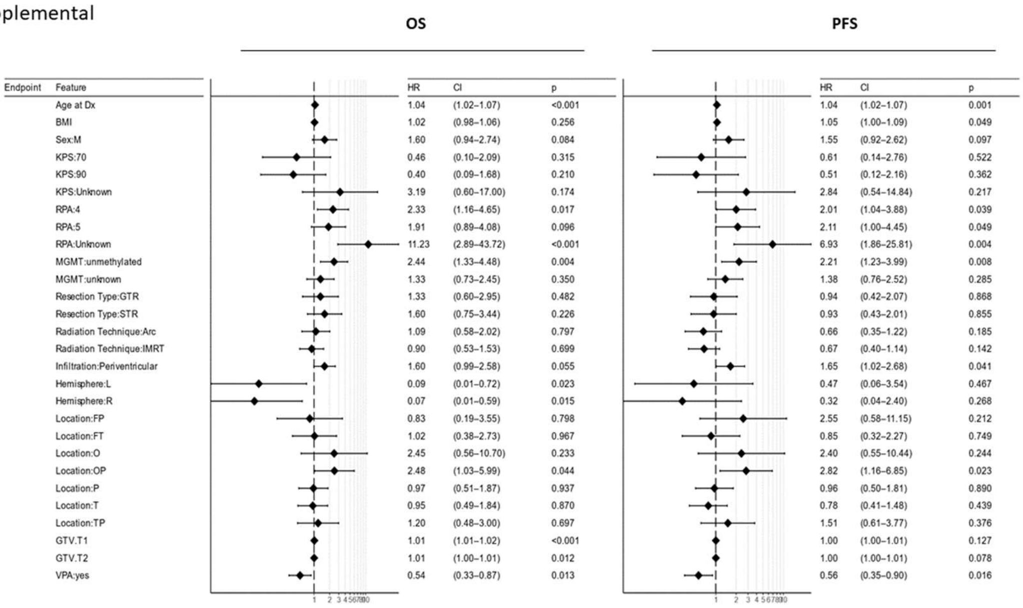

Figure S1: Forest plot for covariates for overall survival (OS) and progression free survival (PFS).

```
table(VPA)

 no yes
 53  29
```

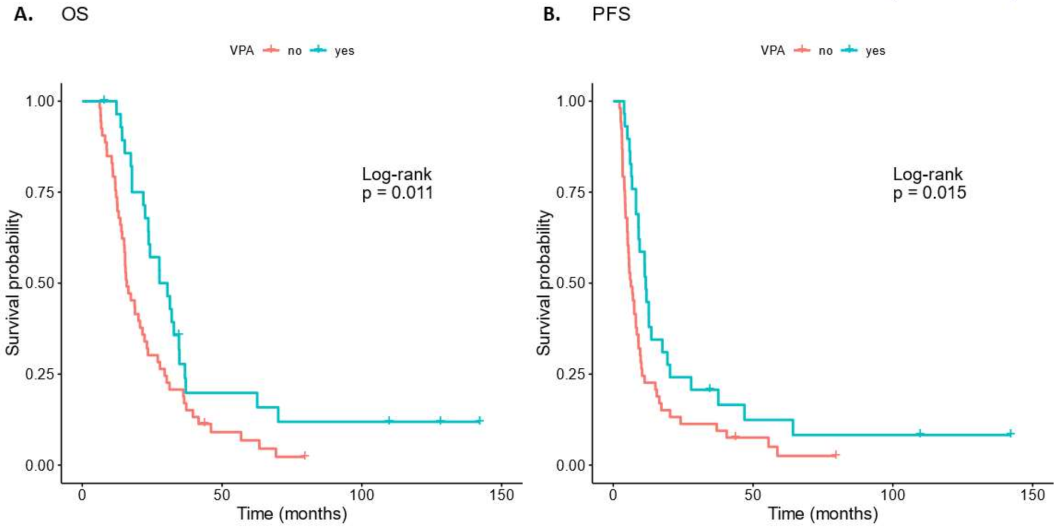

Figure S2: KM curves for OS (A) and PFS (B) for the administration of VPA.

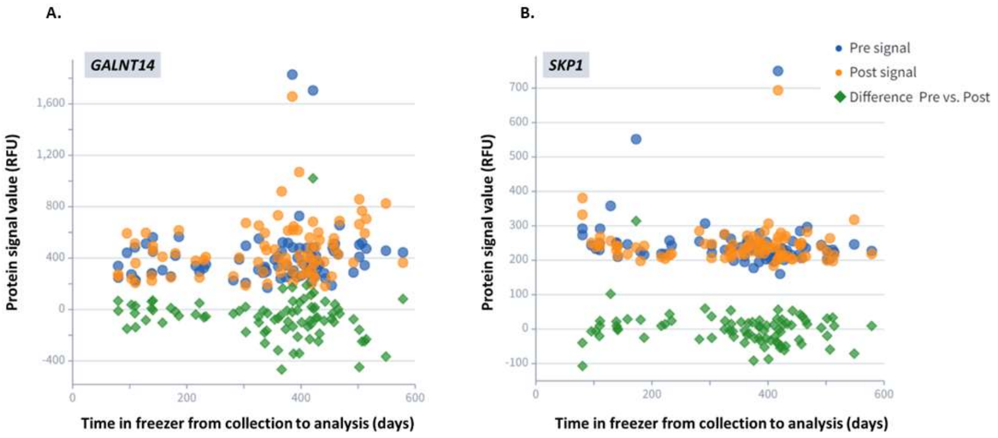

Figure S3: Protein signal vs. time in freezer from collection to analysis in days for.(A) GALNT14, statistically significant for OS and PFS in Cox analysis and survival. (B) SKP1, predictive for response to VPA in prediction model.

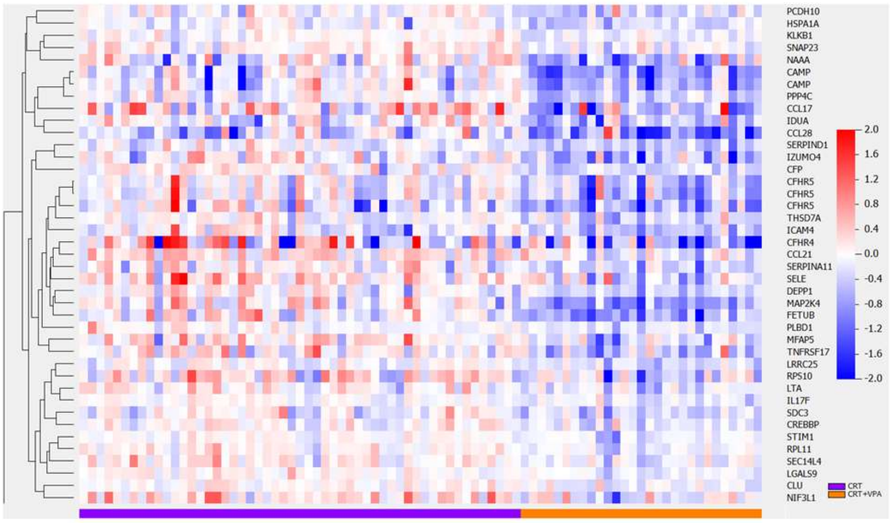

A

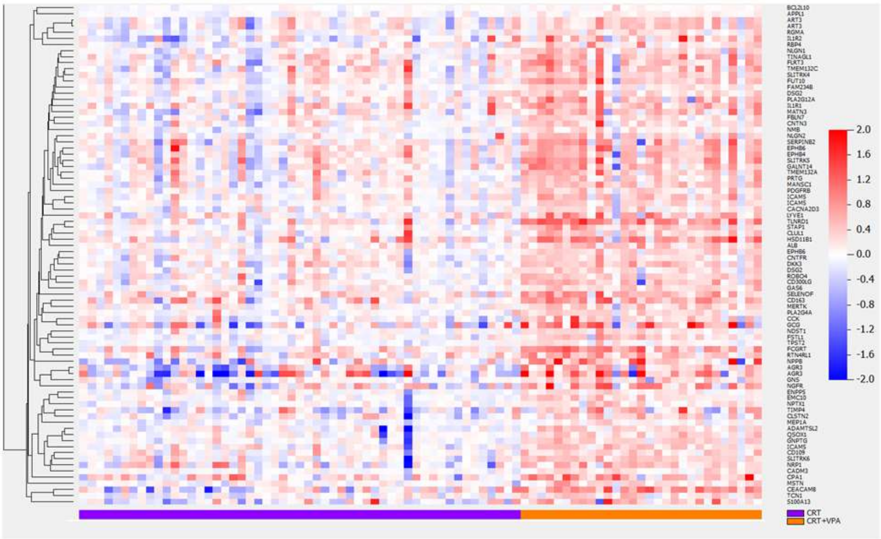

B

Figure S4: Figure 3 displayed as the two separate zoom-in figures with gene names to represent the top (A) and bottom (B) clusters of proteins in the original heatmap.

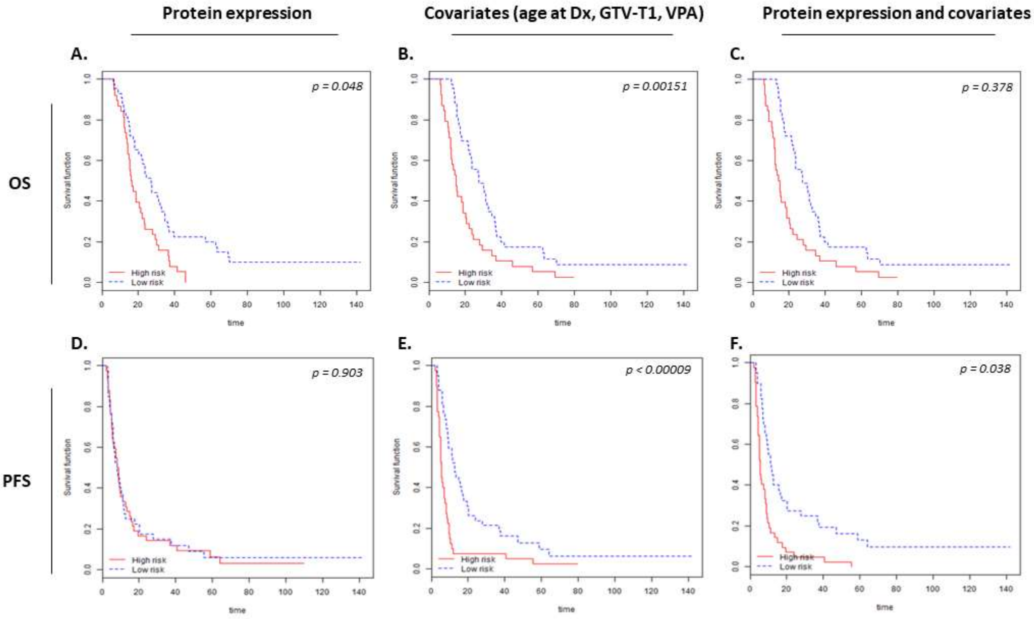

Figure S5: Kaplan Meier curves for survival (OS) (A–C) and progression free survival (PFS) (D–F) with patient population risk stratified (high(red), low (blue)) by protein expression (A,D), covariates (B,E) and combination of protein expression and covariates (C,F) Protein data is based on the results from 123 significantly differentially expressed proteins from class comparison between patients treated with CRT and concurrent VPA vs CRT.

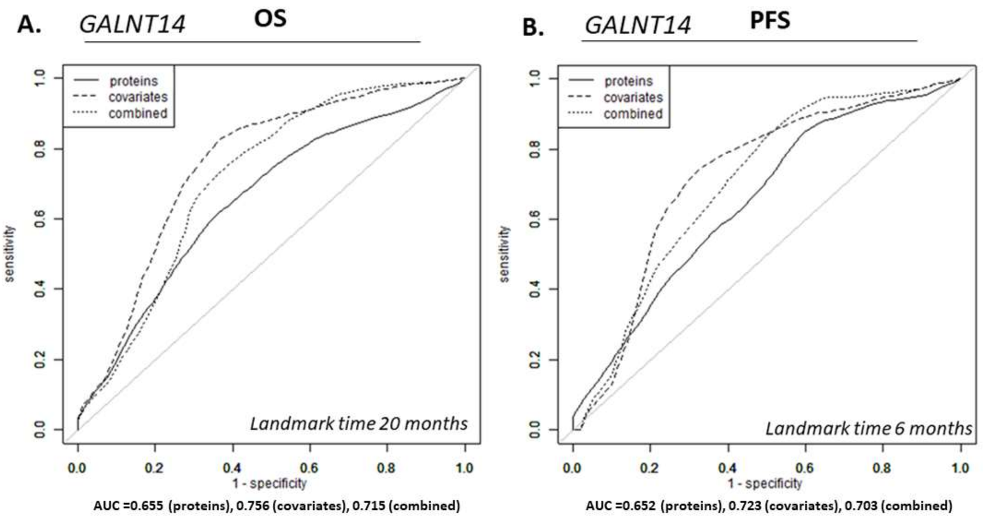

Figure S6: Time-dependent ROC curve for overall survival (OS) (A) and progression free survival (PFS) (B) prediction model based on GALNT14.

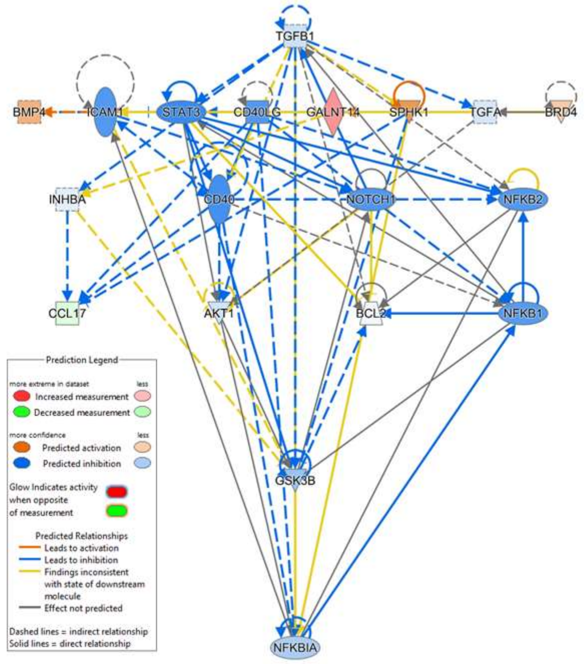

Figure S7: GALNT14 and CCL17, the two proteins associated with OS with FDR 0.104 with potential function between two major signaling pathways driven by TGF and NF-κB. ((QIAGEN Inc., https://www.qiagenbioinformatics.com/products/ingenuitypathway-analysis) accessed 24 July 2023 [71].

Table S1: Patient characteristics table and cox regression analysis of clinical covariates in the VPA cohort.

		OS			PFS		
**Clinical Factors**	***n (%)***	**HR**	**95% CI**	**p-value**	**HR**	**95% CI**	**p-value**
**Age at Diagnosis *mean (SD)***	52.34 (9.60)	*Age as continuous variable*					
Age range	*31-71*	1.035	0.9884 1.085	0.142	1.029	0.9859 1.074	0.192
**Sex**							
**Male**	21 (72.4)	1.631	0.6034 4.408	0.335	1.204	0.4975 2.915	0.68
**Location**							
Periventricular	6 (20.7)	1.456	0.5369 3.951	0.738	1.33	0.4931 3.586	0.573
**Hemisphere**							
Left	15 (51.7)						
Right	14 (48.3)	0.8341	0.373 1.865	0.659	0.7087	0.3245 1.548	0.388
**Resection Type**							
Biopsy	1 (3.4)						
GTR	16 (55.2)	0.2361	0.02741 2.035	0.189	0.3537	0.04313 2.901	0.333
STR	12 (41.4)	0.2224	0.02538 1.949	0.175	0.2651	0.03119 2.253	0.224
**MGMT Status**							
methylated	9 (31.0)						
unmethylated	8 (27.6)	3.186	1.0464 9.702	*0.0414	2.688	0.9445 7.649	0.0639
unknown	12 (41.4)	2.77	0.9895 7.754	0.0524	2.556	0.9541 6.847	0.062
**KPS**		*Baseline 70*					
	90	1.271	0.3733 4.33	0.701	1.037	0.3067 3.507	0.953
60-80	3 (10.3)						
90	12 (41.4)						
100	14 (48.3)						
**RPA**							
RPA 3	9 (31.0)						
RPA 4	17 (58.6)	2.86	1.0093 8.104	*0.048	2.865	1.0365 7.917	*0.0425
RPA 5	3 (10.3)	1.298	0.2609 6.458	0.75	1.086	0.2226 5.297	0.9188
**RT volumes**							
**GTV T1**		*GTV T1 as continuous variable*					
		1.011	0.9944 1.028	0.195	1.01	0.9939 1.027	0.221
<20 cc	11 (37.9)						
20-40 cc	9 (31.0)						
>40 cc	9 (31.0)						
**GTV T2**		*GTV T2 as continuous variable*					
		1.006	0.9981 1.015	0.132	1.003	0.9957 1.011	0.415
<10 cc	0 (0.0)						
10-50 cc	8 (27.6)						
50-100 cc	8 (27.6)						
>100 cc	13 (44.8)						
**Radiation Technique**							
3D	9 (31.0)						
Arc	0 (0.0)						
IMRT	20 (69.0)	0.7431	0.3143 1.757	0.499	0.6184	0.2661 1.437	0.264

Table S2: Cox analysis for overall survival (OS) and progression free survival (PFS) with hazard ratio and p-value for VPA and no VPA patients.

		OS			PFS	
Factors	HR	95% CI	p-value	HR	95% CI	p-value
Age at Diagnosis	1.045	1.019 1.071	***0.000552**	1.041	1.016 1.065	***0.00102**
BMI	1.023	0.9834 1.065	0.256	1.046	1 1.094	***0.0494**
Sex						
Male	1.604	0.9378 2.744	0.0844	1.554	0.9229 2.617	0.0973
KPS						
70	0.4597	0.10095 2.093	0.315	0.6111	0.1355 2.757	0.522
90	0.3967	0.09341 1.685	0.210	0.5127	0.1220 2.155	0.362
unknown	3.1886	0.59814 16.998	0.174	2.8370	0.5424 14.839	0.217
RPA						
RPA 4	2.326	1.1627 4.652	0.017027	2.006	1.037 3.880	***0.03866**
RPA 5	1.908	0.8917 4.081	0.096007	2.114	1.005 4.448	***0.04860**
unknown	11.234	2.8870 43.716	***0.000484**	6.930	1.861 25.806	***0.00391**
MGMT Status						
unmethylated	2.442	1.3320 4.479	***0.00389**	2.212	1.2263 3.990	***0.00835**
unknown	1.335	0.7284 2.445	0.35029	1.385	0.7624 2.515	0.28507
Resection Type						
GTR	1.330	0.6001 2.948	0.482	0.9351	0.4232 2.066	0.868
STR	1.603	0.7467 3.443	0.226	0.9306	0.4311 2.009	0.855
Radiation Technique						
Arc	1.0852	0.5822 2.023	0.797	0.6569	0.3530 1.222	0.185
IMRT	0.9003	0.5287 1.533	0.699	0.6722	0.3958 1.142	0.142
GTV-T1	1.013	1.006 1.02	***0.000346**	1.005	0.9987 1.011	0.127
GTV-T2	1.006	1.001 1.01	0.0121	1.003	0.9966 0.9996	0.0782
Infiltration						
Periventricular	1.598	0.9896 2.58	0.0552	1.652	1.02 2.676	***0.0412**
Hemisphere						
Left	0.08523	0.010143 0.7162	***0.0234**	0.4747	0.06366 3.540	0.467
Right	0.07018	0.008336 0.5909	***0.0145**	0.3207	0.04287 2.398	0.268
Location						
Location FP	0.8268	0.1927 3.548	0.7980	2.5540	0.5850 11.152	0.2124
Location FT	1.0207	0.3821 2.727	0.9674	0.8522	0.3199 2.270	0.7491
Location O	2.4527	0.5623 10.698	0.2325	2.3980	0.5508 10.439	0.2438
Location OP	2.4801	1.0264 5.992	***0.0436**	2.8151	1.1566 6.852	***0.0226**
Location P	0.9742	0.5084 1.867	0.9372	0.9557	0.5034 1.814	0.8897
Location T	0.9462	0.4874 1.837	0.8702	0.7757	0.4074 1.477	0.4395
Location TP	1.1996	0.4798 2.999	0.6971	1.5117	0.6054 3.775	0.3762
VPA						
Yes	0.5355	0.3278 0.8749	***0.0126**	0.5573	0.3461 0.8974	***0.0162**

Table S3: Cancer Hallmark GeneSets associated with Valproic acid (VPA) administration.

GeneList GeneSets	Number of genes	KS permutation p-value	FDR
HALLMARK_EPITHELIAL_MESENCHYMAL_TRANSITION	150/200	0.00009	0.005
HALLMARK_HEDGEHOG_SIGNALING	24/36	0.00031	0.008
HALLMARK_IL6_JAK_STAT3_SIGNALING	74/87	0.00706	0.118
HALLMARK_COAGULATION	112/138	0.01036	0.130
HALLMARK_NOTCH_SIGNALING	19/32	0.01456	0.140
HALLMARK_APICAL_JUNCTION	123/200	0.01674	0.140
HALLMARK_XENOBIOTIC_METABOLISM	123/200	0.02106	0.150
HALLMARK_COMPLEMENT	148/200	0.04952	0.283

Table S4: Results of online sample size calculation tool to calculate the minimum sample size for PFS and OS.

	Input Parameters			*Output*			
	standardized fold change	*Number of proteins*	*prevalence in largest group*		*Minimum sample size in class 1*	*Minimum sample size in class 2*	*Minimum sample size*
PFS	*1.44*	*7289*	*0.646*	*Tolerance=0.05*	*37*	*21*	*58*
				Tolerance=0.10	*29*	*16*	*45*
OS	*1.19*	*7289*	*0.524*	*Tolerance=0.05*	*39*	*35*	*74*
				Tolerance=0.10	*30*	*27*	*57*

**Class 1 is for the group that has the largest prevalence.*

***Conceptually the patients with survival time greater than a landmark time T can be considered the low risk class and those with survival less than T as the high risk class. T should be selected to approximately maximize the difference in survival distributions between the two classes and the prevalence determined accordingly.*
