# Supplementary material for: Revisiting Concurrent Radiation Therapy, Temozolomide, and the Histone Deacetylase Inhibitor Valproic Acid for Patients with Glioblastoma—Proteomic Alteration and Comparison Analysis with the Standard-of-Care Chemoirradiation
Source: Biomolecules. 2023 Oct 10;13(10):1499. doi: 10.3390/biom13101499 (PMC10604983; doi:10.3390/biom13101499)
Supplement: Supplementary file 1 [file biomolecules-13-01499-s001.zip › Supplementary file S1.pdf]

Here is the general formula for the Cox Proportional-Hazards Regression Model:

In its most basic form, for a single predictor variable, the model is expressed as:

$\lambda(t) = \lambda0(t) * \exp(\beta * X)$

where

$\lambda(t)$ represents the hazard rate at time t for an individual.

$\lambda0(t)$ is the baseline hazard function, which represents the hazard when all predictor variables (X) are equal to 0.

β is the coefficient associated with the predictor variable X.

X is the value of the predictor variable.

For multivariate Cox models, the formula extends to:

$\lambda(t) = \lambda0(t) * \exp(\beta1 * X1 + \beta2 * X2 + ... + \beta n * Xn)$

where

$\lambda(t)$ is the hazard rate at time t for an individual.

$\lambda0(t)$ is the baseline hazard function.

$\beta1, \beta2, ..., \beta n$ are the coefficients associated with each of the predictor variables X1, X2, ..., Xn.

The formula for estimating the Kaplan-Meier survival probability at a specific time point, say "t," is as follows:

$S(t) = \Pi [1 - (d_i / n_i)]$

where

S(t) represents the estimated survival probability at time "t."

Π denotes the product over all time points "t_i" where "t_i" is less than or equal to "t."

d_i represents the number of events (e.g., deaths or failures) that occurred at time "t_i."

n_i represents the number of subjects or patients who were at risk (i.e., still under observation and had not experienced the event) just before time "t_i."
