# Supplementary material for: Revisiting Concurrent Radiation Therapy, Temozolomide, and the Histone Deacetylase Inhibitor Valproic Acid for Patients with Glioblastoma—Proteomic Alteration and Comparison Analysis with the Standard-of-Care Chemoirradiation
Source: Biomolecules. 2023 Oct 10;13(10):1499. doi: 10.3390/biom13101499 (PMC10604983; doi:10.3390/biom13101499)
Supplement: Supplementary file 1 [file biomolecules-13-01499-s001.zip › Supplementary file S4.pdf]

**Symbol**	**Name**	**Relevance to cancer**	**Relevance in GBM**
**Prognostic**			
CCL17	C-C motif chemokine ligand 17	Yes	Yes (refs already in discussion)
GALNT14	Polypeptide N-acetylgalactosaminyltransferase 14	Yes	Yes (refs already in discussion)
CTSV	Cathepsin V	Yes[73]	Possible[74]
ACP6	Acid phosphatase 6, lysophosphatidic	Yes[75]	Possible[76]
BMP6	Bone morphogenetic protein 6	Yes[77]	Yes[78]
MSTN	Myostatin	Yes[79]	Yes[80]
SLITRK6	SLIT and NTRK like family member 6	Yes[81]	Yes (may be CNS and/or tumor defining)[82]
ICAM4	Intercellular adhesion molecule 4 (Landsteiner-Wiener blood group)	Yes[83]	Yes[84]
NPTX1	Neuronal pentraxin 1	Yes[85]	Yes (neurodegeneration)[86]
SLITRK5	SLIT and NTRK like family member 5	Yes[87]	Yes (may be CNS and/or tumor defining)[82]
**Potentially predictive**			
PLCD3	Phospholipase C Delta 3	Yes	Yes (refs already in discussion)
SKP1	S-phase kinase-associated protein 1	Yes	Yes (refs already in discussion)
VARS1	Valyl-tRNA synthetase 1	Not defined	Yes (seizures)[88]
CYREN	Cell cycle regulator of NHEJ	Yes[89]	Yes (management) [90]
KIR2DL4	Killer cell immunoglobulin-like receptor 2DL4	Yes[91]	Yes (immune microenvironment)[92]
